# Diseases of the Breast

**Published:** 1895-03

**Authors:** 


*Diseases of the Breast.* By W. ROGER WILLIAMS. Pp. vii., 572. London: JOHN BALE & SONS. 1894.

We believe this work to be one of the most thorough and trustworthy monographs recently published. It is complete in every way, and the information given is well up to date. The labour of its production must have been very great, but the result is so satisfactory that it is well rewarded. From the pathological and clinical aspects it is equally good. Of course, no book of this kind can be written wholly on the experience of the writer, or it would be conspicuously incomplete; but the author of this monograph has illustrated it throughout with cases under his own observation, and his own views are frequently to be found, based generally on careful and elaborate investigation. So much for the general character of the work; but before considering the subjects treated of, we ought perhaps to draw attention to the fact that it is written with special reference to cancer of the breast. The first two chapters are devoted to the origin and morphology of the breast, and then follows an account of mammary abnormalities. We turn to the chapter on axillary mammary structures with much interest in connection with Mr. Paul's recent case of scirrhus in the axilla arising in connection with long convoluted tubes which opened into the hair-follicles and were associated with acini like those of the mammary gland in their deeper parts; and we find that the author describes long convoluted (?) sweat-glands of unusual length and penetrating more deeply than ordinary sweat-glands, as present in the axilla, and reference is made to the fact that Creighton has seen neoplasms arise from them in dogs. Mr. Roger Williams describes axillary swellings which secrete milk, and gives several illustrative cases, but he regards them as sequestrations of the axillary process of the breast. It seems to us that if milk is discharged from the surface of such lumps either through one small opening or several very minute ones (as described by the author), and not through the nipple, they must rather be regarded as supernumerary milk-secreting structures. Of such a nature seem to be the "axillary lumps" of Champneys.

The pathology of cancer of the breast is well described and well illustrated, and that by a writer who has evidently given very careful consideration to the general pathology of malignant growths. He cannot accept the protozoön theory of the causation of cancer, and he gives us several very powerful arguments against its probability. In this, we must say, we heartily agree with him. We have looked in vain for some notice of a controversy which has lately been going on with reference to what has been called the precancerous stage of cancer of the breast, and the question of diffusion of the growth in the breast rather by a general tendency to changes in the epithelium than to spread of the disease by means of the lymphatics. Possibly the author sees no evidence in favour of this view, an opinion which we hold ourselves; but still he might have given us the arguments on both sides, especially as it has been much debated both on the Continent and in this country of late years.

The chapter on so-called villous duct cancers will be full of information to many. Our knowledge of so-called duct cancer is just ripening; we are getting a clearer knowledge of what we mean by duct cancer, though the term still covers several dissimilar growths. This Mr. Roger Williams points out, and he shows that many growths called duct cancer are not cancer at all, but cystic papillomata. He also describes many growths as tubular cancers—tumours distinct from the ordinary scirrhous type or acinous cancer. We consider that the growths referred to under this heading described in the Transactions of the Pathological Society might more accurately have been also included under the first group of villous papillomata.

It is, perhaps, to the clinical portion of the work that most surgeons will turn with the greatest interest, and their expectations will not be disappointed. We are very glad to see that Mr. Roger Williams is in thorough agreement with Banks, Watson Cheyne, and Gross as to the advisability of a very free removal of the breast, the pectoral fascia, and the glands in the axilla, together with the whole of its fatty contents, in every case of cancer, whether enlarged glands can be felt in the axilla or not. Again and again in connection with this operation have we been struck with the fact that infected glands were discovered in the fatty tissue removed, which would inevitably have been left behind had not the axilla been cleared out, even though no enlarged glands could be felt before operation. That most important topic, the differential diagnosis of cancer, is ably discussed. The author mentions chronic mastitis and cold abscess as the conditions he has seen most frequently mistaken for it, and describes graphically a clinical lecture given by the celebrated surgeon of Paris, Verneuil, on a young woman with a swelling in her breast, in which he pointed out why he thought it cancer rather than chronic abscess. Verneuil then proceeded to amputate the breast, when an abscess was discovered. Mr. Roger Willlams also mentions tense cysts as liable to be mistaken for cancer. We should certainly have included them amongst the conditions most frequently mistaken for cancer, for we have several times seen the mistake only rectified by an exploratory incision. The subject of tubercular disease of the breast, about which very little was known in this country until within recent years, is well and fully treated of, and we are reminded that, though we in this country have been so backward in recognising it, Velpeau, as long ago as 1854, gave a graphic description of the disease, from which Mr. Roger Williams's account is largely taken.

